# Associations of Atopobium, Garderella, Megasphaera, Prevotella, Sneathia, and Streptococcus with human papillomavirus infection, cervical intraepithelial neoplasia, and cancer: a systematic review and meta-analysis

**DOI:** 10.1186/s12879-025-10851-4

**Published:** 2025-05-16

**Authors:** Yan Peng, Qin Tang, Shiming Wu, Chengzhi Zhao

**Affiliations:** Department of Obstetrics and Gynecology, Chongqing Health Center for Women and Children, Longshan Road 120, Yubei District, Chongqing, 400010 People’s Republic of China

**Keywords:** Human papillomavirus, Cervical intraepithelial neoplasia, Cervical cancer, Vaginal microbiota, Meta-analysis

## Abstract

**Background:**

Lactobacillus spp. depleted and high diversity of vaginal microbiota is closely related to human papillomavirus infection and cervical cancer. However, the role of other microbial communities in human papillomavirus infection and cervical cancer is still unclear.

**Objective:**

This study aims to systematically review the existing literature and perform a meta-analysis to statistically evaluate the relationship between vaginal microbiota, human papillomavirus infection, cervical intraepithelial neoplasia, and cervical cancer at the genus level.

**Methods:**

A comprehensive search of PubMed, Web of Science, and Embase databases was conducted to identify relevant studies. We synthesized data on the relative abundance of specific bacterial species associated with human papillomavirus status and cervical lesions. SPSS 25.0 was used to compare relative abundance among multiple groups.

**Results:**

The meta-analysis included 17 observational studies published between 2019 and 2023, involving 2014 participants from Asia, North America, and Africa. We found that specific vaginal microorganisms, such as Gardnerella, Prevotella, Sneathia, and Streptococcus, showed increased relative abundance with the severity of cervical lesions in human papillomavirus-negative, human papillomavirus-positive, cervical intraepithelial neoplasia, and cervical cancer patients. However, no statistically significant differences were found in that regard. Notably, Prevotella was significantly more abundant in cervical cancer patients compared to human papillomavirus-negative individuals. Sneathia was also found to be more abundant in cervical intraepithelial neoplasia and cervical cancer patients.

**Conclusions:**

The specific vaginal microbial species are associated with human papillomavirus infection status and the severity of cervical lesions that may have significant implications for the prevention and treatment strategies of cervical cancer.

## Introduction

Cervical cancer (CC) is the fourth most common malignant tumor among women worldwide, with a persistently high incidence and mortality rate globally. Approximately 660,000 new cases and 350,000 deaths occur every year, showing a trend of gradual increase and affecting younger populations, particularly in developing countries [1]. Despite the significant reduction in CC incidence in some countries due to the promotion of vaccination and screening programs, the disease remains prevalent in resource-limited areas [2].

The occurrence of CC is closely related to human papillomavirus (HPV) infection, particularly HPV types 16 and 18, which account for approximately 70% of CC cases [3]. Although HPV infection is widespread, only a small proportion of infections progress to CC, indicating that other factors also play significant roles in the pathogenesis of this disease. Abnormal immune function, genetic susceptibility, and dysregulation of the microenvironment are potential factors influencing the outcomes of infection [4–6]. In recent years, growing evidence suggests that microorganisms play a critical role in the occurrence and development of cancer. Persistent infections caused by dysregulated microorganisms create a pro-inflammatory environment that promotes the occurrence of cancer [7–11]. Statistics indicate that approximately 15% of new cancer cases worldwide each year are attributable to infections [12]. Currently, six viruses and one bacterium have been definitively identified as the cause of cancer, namely HPV, hepatitis B virus (HBV), hepatitis C virus (HCV), Epstein-Barr virus (EBV), human T-cell lymphotropic virus type 1, Kaposi’s sarcoma-associated herpesvirus, and Helicobacter pylori [13].

With the advancement of human microbiome research, the role of microorganisms in human health and disease has gradually been recognized. The interactions between microorganisms and their hosts are complex and diverse, exerting widespread effects on physiological and pathological processes in the human body. Particularly in the field of cancer, microorganisms have been found not only to participate in the occurrence and development of cancer but also to potentially influence the effectiveness of treatment [14]. Specifically, altering the composition of the microbiome, such as through the use of probiotics, prebiotics, or microbial transplantation, may affect the tumor microenvironment and immune responses, thereby impacting the occurrence, progression, and therapeutic efficacy of cancer. Supplementation with specific probiotics has been shown to improve the response rate of cancer patients to immune checkpoint inhibitors [15]. Similarly, in CC, changes in the composition and diversity of the reproductive tract microbiota may influence the persistence of HPV infection and the progression of precancerous lesions [16]. Literature explicitly states that the absence of Lactobacillus can promote the development of CC [17]. However, other microbial communities beyond Lactobacillus also play a role in the occurrence and progression of CC, but research on these microorganisms is relatively limited.

Bacterial vaginosis (BV) is a common condition characterized by an imbalance in the vaginal microbiota (VMB), typically associated with a decrease in beneficial Lactobacillus species and an increase in various anaerobic and facultative bacteria [18]. Multiple meta-analyses have demonstrated a close association between BV and CC, with a positive correlation to the severity of cervical lesions (such as HPV infection, cervical intraepithelial neoplasia (CIN), and CC) [19–21]. Studies suggest that microorganisms associated with BV, such as Fusobacterium, Leptotrichia, Prevotella, and Porphyromonas, are linked to gynecological or other cancers. The carcinogenic substances they produce, along with their potential impact on chronic inflammation and immune modulation, increase the risk of developing tumors in women with BV [22]. In addition, BV may create a more favorable environment for the infection and integration of HPV into host cells by disrupting the vaginal barrier function, thereby promoting the persistence of HPV [23].

It is suggested antibiotics or antimicrobial agents targeting specific microorganisms may also represent a new strategy for cancer treatment. However, research on specific harmful bacteria associated with CC remains limited. With the development of high-throughput sequencing technologies, such as 16 S rRNA gene sequencing, metagenomic sequencing, and single-cell sequencing, have provided powerful tools to understand the relationship between microorganisms and cancer. These technologies not only facilitate the identification of specific microbial species associated with CC but also reveal changes in microbial community structure and function, providing new biomarkers for disease diagnosis and treatment. Therefore, this study aims to systematically review the existing literature and perform a meta-analysis to statistically evaluate the relationship between VMB, HPV infection, CIN, and CC at the genus level.

## Methods

This study follows the PRISMA guidelines for systematic reviews and meta-analyses [24] and has been registered on PROSPERO (Registration Number: CRD42024583392). Since it does not involve human participants or human-related data, ethical approval is not required.

### Eligibility criteria

Inclusion criteria in this study were as follows: (1) Type of study included: observational studies; (2) Study population: healthy population, HPV-infection, CIN, and CC patients; (3) Outcome measures: analysis of VMB with sequencing data. Exclusion criteria in this study were as follows: (1) Studies involving human cell lines or animal models; (2) Pregnant women or patients under the age of 18; (3) Patients with immunodeficiency disorders; (4) Non-English articles, reviews, or comments; (5) Lack of original data.

### Search strategy

The electronic databases PubMed, Web of science, and Embase were searched from inception to July 2024 by using search terms in combination with both MeSH terms and free text for ((Human Papillomavirus Virus) OR (HPV) OR (Papillomavirus Virus, Human) OR (Virus, Human Papillomavirus) OR (Human Papilloma Virus) OR (Human Papilloma Viruses) OR (Papilloma Virus, Human) OR (Virus, Human Papilloma) OR (Human Papillomavirus) OR (Human Papillomaviruses) OR (HPV Human Papillomavirus) OR (HPV Human Papillomaviruses) OR (Human Papillomaviruses, HPV) OR (Human Papillomavirus, HPV) OR (HPV, Human Papillomavirus Viruses)) AND ((Microbiota) OR (VMB) OR (Microbiotas) OR (Microbial Community) OR (Community, Microbial) OR (Microbial Communities) OR (Microbial Community Composition) OR (Community Composition, Microbial) OR (Composition, Microbial Community) OR (Microbial Community Compositions) OR (Microbiome) OR (Microbiomes) OR (Human Microbiome) OR (Human Microbiomes) OR (Microbiome, Human) OR (Microbial Community Structure) OR (Community Structure, Microbial) OR (Microbial Community Structures)).

### Study selection and data extraction

Three researchers (Yan Peng, Qin Tang, and Shiming Wu) independently screened the literature and extracted data according to pre-designed inclusion and exclusion criteria. Firstly, the titles were reviewed, and if relevant, the abstracts and full texts were examined. The inclusion of all literature was decided jointly by the three researchers. In case of disagreements, resolution was achieved through discussion or consultation with a senior researcher (Chengzhi Zhao). EndNote X9 was used to manage and screen the literature. The three researchers (Yan Peng, Qin Tang, and Shiming Wu) extracted data according to a pre-designed data extraction form, including (1) Basic characteristics of the included studies: publication year, author names, sample size, country, and study period; (2) Baseline characteristics of the study subjects: age, pregnancy status, immunodeficiency status, HPV status, and cervical pathological status (based on cytology or histology); (3) Exposure factors: VMB sequencing data, with the relative abundance of dominant bacterial species calculated.

### Assessment of risk of bias

Two researchers (Yan Peng and Chengzhi Zhao) independently assessed the risk of bias for each included study using the Newcastle-Ottawa Scale, a tool for evaluating the quality of case-control studies in meta-analyses [25]. The scale includes four items related to the selection of study participants, one item on comparability between groups, and three items on the measurement of exposure factors. Except for the comparability item, which can score up to two points, all other items can score a maximum of one point. The total score ranges from 0 to 9, with higher scores indicating a lower risk of bias.

### Data synthesis

The main outcomes of the study are the relationships between VMB and HPV negative, HPV positive, CIN, and CC, which are summarized separately in the meta-analysis. Other factors were not analyzed. The cumulative relative abundance of different dominant bacteria in each study was summarized weighted and expressed as a percentage. Due to the inevitable heterogeneity between studies, a random-effects model was used to combine the data, and effect sizes (rates) and the corresponding 95% confidence intervals were calculated using Stata 17.0. Heterogeneity between study results was analyzed using Cochran’s Q test, and the degree of heterogeneity was determined with I². If the Q test results were statistically significant (*P* < 0.05), it indicated the presence of heterogeneity between the effect sizes of the included studies. If I² ≥ 50%, it suggested substantial heterogeneity between the effect sizes of the included studies. When heterogeneity tests indicate considerable heterogeneity among the included studies, further analysis of the source of heterogeneity and sensitivity analysis becomes more meaningful.

Data analysis was performed using SPSS 25.0 statistical software. The chi-square test or Fisher’s exact test was used for comparison of rates among multiple groups, with *P* < 0.05 considered statistically significant.

## Results

### Study selection

**Fig. 1:**
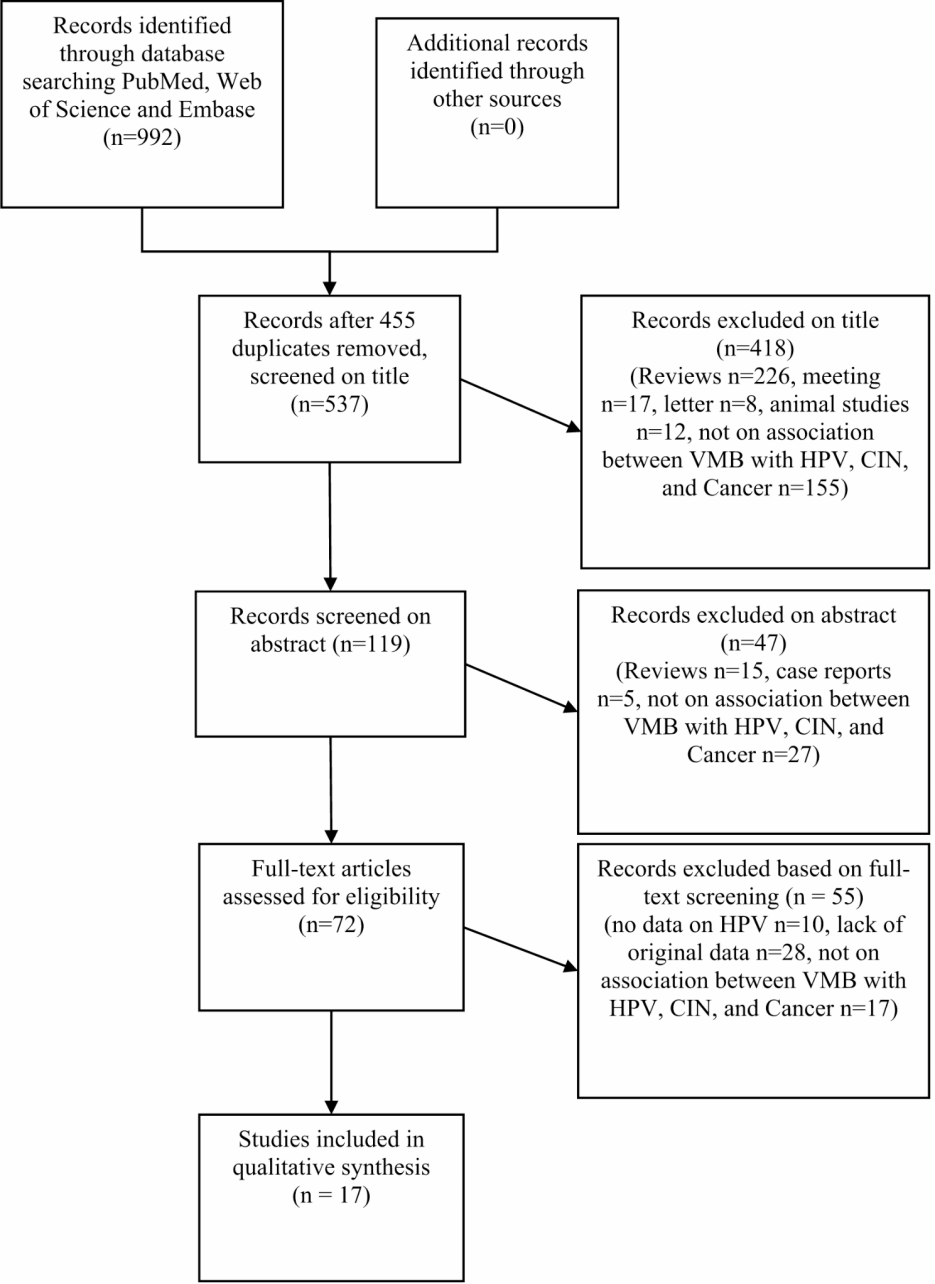
The study flowchart

A total of 992 relevant articles were initially identified. After excluding 455 duplicates, 465 articles were excluded based on the inclusion and exclusion criteria after reading the titles and abstracts. Subsequently, the full texts of 72 articles were reviewed, and finally, 17 studies were included, all of which were observational studies. The literature selection process and results are shown in Fig. 1.

### Study characteristics

A total of 17 studies were included, published between 2019 and 2023. Among these 17 studies, 15 were conducted in Asia [26–40], 1 in North America [41], and 1 in Africa [42]. A total of 2014 participants were involved, including 557 healthy individuals, 839 HPV-positive cases, 415 CIN cases, and 203 CC cases, with an average age range of 19 to 75 years. All studies performed 16 S rRNA sequencing of the VMB of the included participants and measured the relative abundance of dominant bacteria. The basic characteristics of the included studies are shown in Table 1.

**Table 1:** Study characteristics of all 17 studies included in the systematic review

Study	Country	Age	Study size	Study design	Primary outcomes	Microbial sampling	Microbial Assessment
Chao 2019 [26]	China	20–65 years	151	Cross-sectional	HPV-negative, HPV-positive	vagina	Amplification of the bacterial 16 S rRNA V4 gene region; Illumina sequencing
Chao 2020 [27]	China	30–55 years	190	Cross-sectional	HPV-negative, HPV-positive	vagina	Amplification of the bacterial 16 S rRNA V4 gene region; Illumina sequencing
Chen 2020 [28]	China	30–65 years	229	Cross-sectional	HPV-negative, HPV-positive, CIN, Cancer	vagina	Amplification of the bacterial 16 S rRNA V3–4 hypervariable fragments gene region; Illumina MiSeq sequencing
Chorna 2020 [29]	Puerto Rico	not mentioned	19	Cross-sectional	HPV-negative, HPV-positive	vagina	Using the Quantitative Insights into Microbial Ecology to analyze 16 S rRNA genes
Wei 2020 [30]	China	20–49 years	60	Cross-sectional	HPV-negative, HPV-positive	vagina	Amplification of the bacterial 16 S rRNA V3–4 hypervariable fragments gene region; Illumina MiSeq sequencing
Wu 2020 [31]	China	19–50 years	16	Cross-sectional	HPV-negative, HPV-positive, CIN	vagina	Amplification of the bacterial 16 S rRNA V3–4 gene region; Illumina MiSeq sequencing
Xie 2020 [32]	China	25–39 years	62	Cross-sectional	HPV-negative, CIN, Cancer	vagina	Amplification of the bacterial 16 S rRNA V4 gene region; Illumina Novaseq sequencing
Yang 2020 [33]	China	25–42 years	52	Cross-sectional	HPV-negative, HPV-positive	vagina	Metagenomic Sequencing
Chao 2021 [34]	China	28–49 years	169	Cross-sectional	HPV-positive, CIN	vagina	Amplification of the bacterial 16 S rRNA V4 gene region; Illumina sequencing
Kawahara 2021 [35]	Japan	24–48 years	41	Cross-sectional	CIN	cervicovagina	Amplification of the bacterial 16 S rRNA V3–4 gene region; Illumina MiSeq sequencing
Zhang 2021 [36]	China	28–43 years	68	Cross-sectional	HPV-negative, HPV-positive, Cancer	vagina	Amplification of the bacterial 16 S rRNA V3–4 gene region; Illumina MiSeq sequencing
Mei 2022 [37]	China	21–64 years	70	Cross-sectional	HPV-negative, HPV-positive	vagina	Amplification of the bacterial 16 S rRNA V3–4 gene region; Illumina MiSeq sequencing
Xia 2022 [38]	China	22–47 years	135	Cross-sectional	HPV-negative, HPV-positive, CIN	vagina	Amplification of the bacterial 16 S rRNA V3–4 gene region; Illumina sequencing
Li 2023 [39]	China	29–65 years	317	Cross-sectional	HPV-negative, HPV-positive, CIN, Cancer	vagina	Amplification of the bacterial 16 S rRNA V3–4 gene region; Illumina MiSeq sequencing
Liu 2023 [40]	China	28–43 years	312	Cross-sectional	HPV-positive, CIN	vagina	Amplification of the bacterial 16 S rRNA V4 gene region; Illumina Novaseq6000 sequencing
Ma 2023 [41]	China	20–75 years	63	Cross-sectional	CIN, Cancer	vagina	Amplification of the bacterial 16 S rRNA; Illumina sequencing
Teka 2023 [42]	Ethiopia	not mentioned	60	Cross-sectional	Cancer	cervicovagina	Amplification of the bacterial 16 S rRNA V4 gene region; Illumina MiSeq sequencing

### Quality assessment

Table 2 lists the detailed results of the bias assessment according to the Newcastle-Ottawa Scale. Studies with a score of 7 or higher are considered high-quality studies (low risk of bias). Eight studies had a bias risk score of 9, seven studies had a bias risk score of 8, and the remaining two studies had a score of 7.

**Table 2:** Newcastle-Ottawa scale for risk of bias assessment

Study	Selection				Case-control comparability	Exposure			Total score
	Case definition	Case representativeness	Selection of controls	Definition of controls		Ascertainment of exposure	Same ascertainment for cases and controls	Nonresponse rate	
Chao 2019	1	1	1	1	2	1	1	1	9
Chen 2020	1	1	1	1	2	1	1	0	8
Chao 2020	1	1	1	1	1	1	1	0	7
Chorna 2020	1	1	1	1	2	1	1	1	9
Wei 2020	1	1	1	1	2	1	1	1	9
Wu 2020	1	1	1	1	1	1	1	1	8
Xie 2020	1	1	1	1	2	1	1	0	8
Yang 2020	1	1	1	1	1	1	1	1	8
Chao 2021	1	1	1	1	2	1	1	1	9
Kawahara 2021	1	1	1	1	2	1	1	1	9
Zhang 2021	1	1	1	1	2	1	1	0	9
Mei 2022	1	1	1	1	1	1	1	1	8
Xia 2022	1	1	1	1	2	1	1	1	9
Li 2023	1	1	1	1	1	1	1	0	7
Liu 2023	1	1	1	1	1	1	1	1	8
Ma 2023	1	1	1	1	2	1	1	0	8
Teka 2023	1	1	1	1	2	1	1	1	9

### Synthesis of results

#### Atopobium with HPV, CIN, and CC

**Fig. 2:**
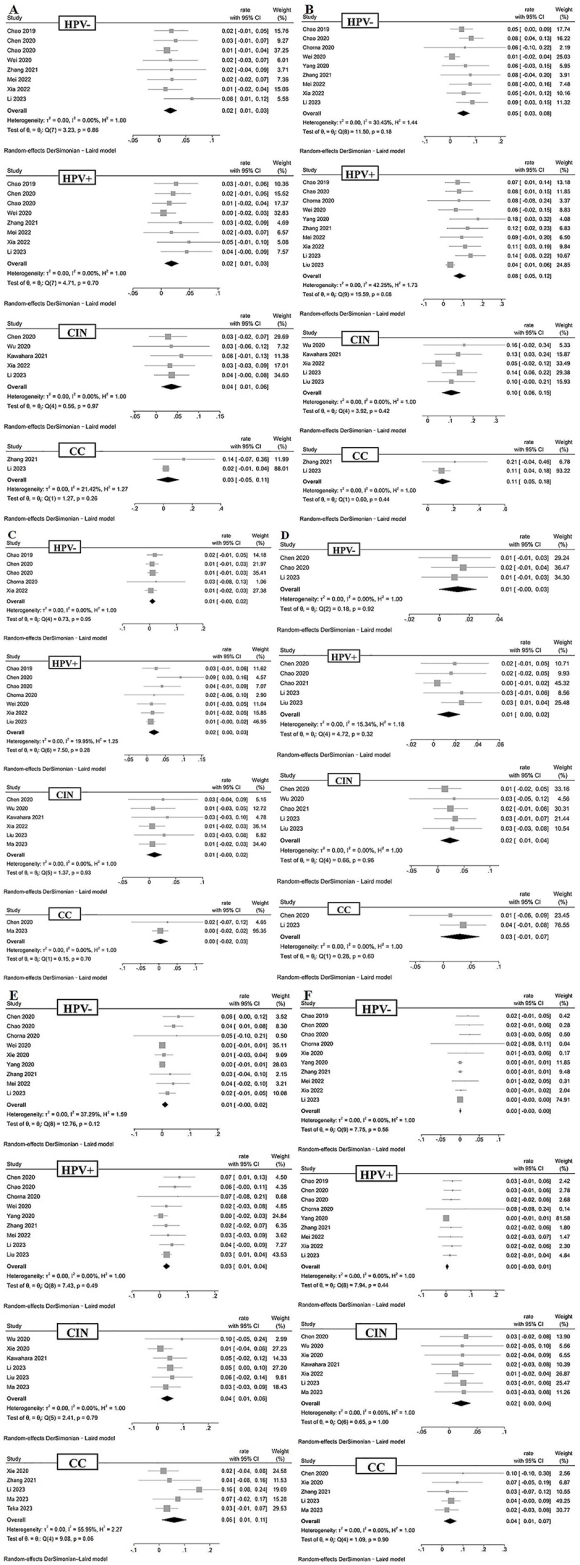
Forest plots between Atopobium (**A**), Garderella (**B**), Megasphaera (**C**), Streptococcus (**D**), Prevotella (**E**), Sneathia (**F**), and human papillomavirus negative (HPV-), human papillomavirus positive (HPV+), cervical intraepithelial neoplasia (CIN), and cervical cancer (CC)

**Fig. 3:**
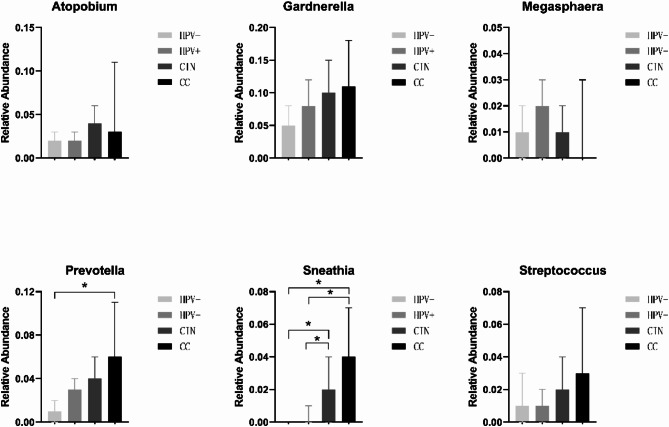
Comparison of the relative abundance of Atopobium, Garderella, Megasphaera, Streptococcus, Prevotella, and Sneathia among different groups. Human papillomavirus negative (HPV-), human papillomavirus positive (HPV+), cervical intraepithelial neoplasia (CIN), and cervical cancer (CC), *P<0.05

As shown in Fig. 2A, eight studies (*n* = 499) reported the relative abundance of Atopobium in the VMB of HPV-negative healthy individuals. The pooled analysis resulted in an overall rate of 2% (95% CI = 0.01–0.03), with very low heterogeneity between studies (I²=0%). Eight studies (*n* = 436) reported the relative abundance of Atopobium in the VMB of HPV-positive individuals, with the pooled analysis showing an overall rate of 2% (95% CI = 0.01–0.03) and very low heterogeneity between studies (I²=0%). Five studies (*n* = 221) reported the relative abundance of Atopobium in the VMB of CIN patients, with the pooled analysis showing an overall rate of 4% (95% CI = 0.01–0.06), and very low heterogeneity between studies (I²=0%). Two studies (*n* = 89) reported the relative abundance of Atopobium in the VMB of CC patients, with the pooled analysis showing an overall rate of 3% (95% CI=-0.05-0.11) and low heterogeneity between studies (I²=21.42%). There was no significant difference in the relative abundance of Atopobium among the groups (*P* > 0.05) (Fig. 3).

#### Garderella with HPV, CIN, and CC

As shown in Fig. 2B, nine studies (*n* = 464) reported the relative abundance of Gardnerella in the VMB of HPV-negative healthy individuals. The pooled analysis resulted in an overall rate of 5% (95% CI = 0.03–0.08), with low heterogeneity between studies (I²=30.43%). Ten studies (*n* = 675) reported the relative abundance of Gardnerella in the VMB of HPV-positive individuals, with the pooled analysis showing an overall rate of 8% (95% CI = 0.05–0.12) and low heterogeneity between studies (I²=42.25%). Five studies (*n* = 203) reported the relative abundance of Gardnerella in the VMB of CIN patients, with the pooled analysis showing an overall rate of 10% (95% CI = 0.06–0.15), and very low heterogeneity between studies (I²=0%). Two studies (*n* = 89) reported the relative abundance of Gardnerella in the VMB of CC patients, with the pooled analysis showing an overall rate of 11% (95% CI = 0.05–0.18), and very low heterogeneity between studies (I²=0%). There was no significant difference in the relative abundance of Gardnerella among the groups (*P* > 0.05) (Fig. 3).

#### Megasphaera with HPV, CIN, and CC

As shown in Fig. 2C, five studies (*n* = 336) reported the relative abundance of Megasphaera in the VMB of HPV-negative healthy individuals. The pooled analysis resulted in an overall rate of 1% (95% CI = 0.00-0.02), with very low heterogeneity between studies (I²=0%). Seven studies (*n* = 580) reported the relative abundance of Megasphaera in the VMB of HPV-positive individuals, with the pooled analysis showing an overall rate of 2% (95% CI = 0.00-0.03) and low heterogeneity between studies (I²=19.95%). Six studies (*n* = 170) reported the relative abundance of Megasphaera in the VMB of CIN patients, with the pooled analysis showing an overall rate of 1% (95% CI = 0.00-0.02), and very low heterogeneity between studies (I²=0%). Two studies (*n* = 36) reported the relative abundance of Megasphaera in the VMB of CC patients, with the pooled analysis showing an overall rate of 0% (95% CI=-0.02-0.03), and very low heterogeneity between studies (I²=0%). There was no significant difference in the relative abundance of Megasphaera among the groups (*P* > 0.05) (Fig. 3).

#### Streptococcus with HPV, CIN, and CC

As shown in Fig. 2D, three studies (*n* = 278) reported the relative abundance of Streptococcus in the VMB of HPV-negative healthy individuals. The pooled analysis resulted in an overall rate of 1% (95% CI = 0.00-0.03), with very low heterogeneity between studies (I²=0%). Five studies (*n* = 582) reported the relative abundance of Streptococcus in the VMB of HPV-positive individuals, with the pooled analysis showing an overall rate of 1% (95% CI = 0.00-0.02) and low heterogeneity between studies (I²=15.34%). Five studies (*n* = 262) reported the relative abundance of Streptococcus in the VMB of CIN patients, with the pooled analysis showing an overall rate of 2% (95% CI = 0.01–0.04), and very low heterogeneity between studies (I²=0%). Two studies (*n* = 88) reported the relative abundance of Streptococcus in the VMB of CC patients, with the pooled analysis showing an overall rate of 3% (95% CI=-0.01-0.07), and very low heterogeneity between studies (I²=0%). There was no significant difference in the relative abundance of Streptococcus among the groups (*P* > 0.05) (Fig. 3).

#### Prevotella with HPV, CIN, and CC

As shown in Fig. 2E, nine studies (*n* = 428) reported the relative abundance of Prevotella in the VMB of HPV-negative healthy individuals. The pooled analysis resulted in an overall rate of 1% (95% CI = 0.00-0.02), with low heterogeneity between studies (I²=37.29%). Nine studies (*n* = 630) reported the relative abundance of Prevotella in the VMB of HPV-positive individuals, with the pooled analysis showing an overall rate of 3% (95% CI = 0.01–0.04) and very low heterogeneity between studies (I²=0%). Six studies (*n* = 224) reported the relative abundance of Prevotella in the VMB of CIN patients, with the pooled analysis showing an overall rate of 4% (95% CI = 0.01–0.06), and very low heterogeneity between studies (I²=0%). Five studies (*n* = 194) reported the relative abundance of Prevotella in the VMB of CC patients, with the pooled analysis showing an overall rate of 6% (95% CI = 0.01–0.11), and moderate heterogeneity between studies (I²=55.95%). When comparing between groups, the relative abundance of Prevotella in the CC group was significantly higher than in the HPV-negative group (*P* < 0.05) (Fig. 3).

#### Sneathia with HPV, CIN, and CC

As shown in Fig. 2F, ten studies (*n* = 527) reported the relative abundance of Sneathia in the VMB of HPV-negative healthy individuals. The pooled analysis resulted in an overall rate of 0% (95% CI = 0.00–0.00), with very low heterogeneity between studies (I²=0%). Nine studies (*n* = 444) reported the relative abundance of Sneathia in the VMB of HPV-positive individuals, with the pooled analysis showing an overall rate of 0% (95% CI = 0.00-0.01), and very low heterogeneity between studies (I²=0%). Seven studies (*n* = 263) reported the relative abundance of Sneathia in the VMB of CIN patients, with the pooled analysis showing an overall rate of 2% (95% CI = 0.00-0.04), and very low heterogeneity between studies (I²=0%). Five studies (*n* = 143) reported the relative abundance of Sneathia in the VMB of CC patients, with the pooled analysis showing an overall rate of 4% (95% CI = 0.01–0.07), and very low heterogeneity between studies (I²=0%). When comparing between groups, the relative abundance of Sneathia in the CIN and CC groups was significantly higher than in the HPV-negative and HPV-positive groups (*P* < 0.05) (Fig. 3).

## Discussion

The results of this study indicate that specific vaginal microbial species are associated with HPV infection status and the severity of cervical lesions. This provides a new perspective on the pathogenesis of CC and may have significant implications for the prevention and treatment strategies of CC. Firstly, our meta-analysis results show that specific vaginal microorganisms, such as Gardnerella, Prevotella, Sneathia, and Streptococcus, have a relative abundance that increases with the severity of cervical lesions in HPV-negative, HPV-positive, CIN, and CC patients. However, no statistically significant differences were found in that regard. Similarly, although Atopobium and Megasphaera are closely related to cervical lesions, no specific relationship between their abundance and the severity of cervical lesions was observed. Additionally, there were no significant differences in the relative abundance of Atopobium, Gardnerella, Megasphaera, and Streptococcus among the groups. This suggests that these microorganisms may not be major driving factors in the development of CC, or their effects may be masked by other factors.

However, it is worth noting that the relative abundance of Prevotella in CC patients is significantly higher than in the HPV-negative group, which may suggest its potential role in the development of CC. This finding is consistent with previous research results [16, 43–44], which indicate that Prevotella is associated with an increased risk of CC. Specific microbial species may increase the risk of CC through the production of carcinogenic substances, the induction of chronic inflammation, and potential immune modulation effects. Dong [45] conducted a study on 920 women and found that the relative abundance of Prevotella in the vagina of women with persistent HPV16 and HPV18 infections was significantly increased. The overgrowth of Prevotella in the vagina may promote the persistent infection of high-risk HPV through host NF-κB and C-myc signaling pathways, leading to the development of cervical lesions. However, further research is needed to verify this association and explore how Prevotella influences the development of CC.

Secondly, the relative abundance of Sneathia in CIN and CC patients is significantly higher than in the HPV-negative and HPV-positive groups. This result suggests that Sneathia may play a role in the progression of cervical lesions. Sneathia is a microorganism associated with BV, which is related to the imbalance of the vaginal microenvironment. The presence of Sneathia is positively correlated with the diagnostic criteria for BV [46–48]. BV is associated with an increased risk of HPV infection and cervical cancer [19–21]. BV may promote the persistent presence of HPV by disrupting the vaginal barrier function, creating a more favorable environment for HPV infection and integration into host cells [23]. Therefore, the increase of Sneathia may reflect the impact of vaginal microenvironment imbalance on the development of CC. Specifically, Sneathia can adhere to cervical epithelial cells, causing changes in cell morphology and disruption of intercellular contacts [49–50]. Sneathia may increase local inflammation and tissue damage by disrupting the stability and integrity of the cervical epithelial cell, thereby increasing the risk of HPV infection and CC. However, as an emerging pathogen, the role of Sneathia in female health and disease is often underestimated. More attention and research are needed in the future to fully understand its role in disease development and to develop effective diagnostic, treatment, and prevention strategies.

Gardnerella vaginalis is a well-known pathogen associated with BV, it is capable of adhering to vaginal epithelial cells and forming biofilms, which can create a favorable environment for the colonization of other anaerobic bacteria (such as Sneathia, and Prevotella) [46]. Although our meta-analysis did not find a significant difference in the relative abundance of Gardnerella across different groups, its role in BV cannot be neglected. BV is characterized by an imbalance of the vaginal microbiome and a characteristic biofilm formed on the vaginal epithelium, which is initiated and dominated by Gardnerella bacteria, creating an anaerobic environment [51]. Sneathia and Prevotella then thrive in this altered niche, producing enzymes and toxins that cause inflammation and tissue damage. These bacteria interact synergistically, exacerbating dysbiosis and weakening the mucosal barrier. Their combined effects promote chronic inflammation, increase susceptibility to infections like HPV, and may drive the progression of cervical lesions.

Our study also has some limitations. Firstly, due to the limited number of included studies, we were unable to analyze all possible vaginal microorganisms. Additionally, this study did not consider other factors that may influence the composition and function of the VMB, such as the host’s lifestyle, dietary habits, and genetic factors. These variables are known to play significant roles in shaping the microbiota and may also influence the relationship between VMB and cervical diseases. Future studies should aim to control for these factors to provide a more comprehensive understanding of the underlying mechanisms. Similarly, the generalizability of our findings may be limited by the geographic and ethnic composition of the included studies. Of the 17 studies included in our meta-analysis, 15 were conducted in Asian populations. This overrepresentation of a single ethnic group may restrict the applicability of our results to other ethnicities and environments. The VMB can vary significantly across different populations due to genetic, environmental, dietary, and lifestyle differences. Therefore, caution should be exercised when extrapolating our findings to non-Asian populations. Future research should include more diverse cohorts better to understand the global implications of VMB in cervical diseases. Secondly, the heterogeneity between studies may affect the interpretation of the results. Although we used a random effects model to combine the data and performed heterogeneity tests, the sources of heterogeneity still need further exploration. Thirdly, our study did not specifically restrict the definition of HPV to oncogenic (high-risk) types, such as HPV 16 and 18, which are most strongly associated with CC development. Consequently, we were unable to precisely delineate the relationship between high-risk HPV infections and the VMB, which is critical for elucidating their role in CC. Future studies should focus on high-risk HPV types to provide more targeted insights into this relationship. Furthermore, we included all studies related to CIN (both LSIL and HSIL) without further stratification. While this approach provides a comprehensive overview, it may limit the accuracy with which we can interpret the variations in the relative abundance of specific VMB across different stages of cervical lesions. Future studies should consider refining the analysis to specifically investigate the relationship between LSIL, HSIL, and VMB to more accurately elucidate their roles in CC development. Lastly, due to the limitations of the study design, we were unable to determine the causal relationship between the VMB, HPV infection, and CC. Future research should adopt prospective designs with larger sample sizes to validate our findings and explore the relationship between the microorganism and the development of CC.

## Conclusion

In conclusion, our findings emphasize the potential role of specific bacterial species in the VMB in the development of CC and provide a new direction for future research. By further exploring the relationship between the VMB, HPV infection, and CC, we can gain a better understanding of the pathogenesis of CC and develop new prevention and treatment strategies. Future studies need to further investigate the specific mechanisms through which particular microbial species contribute to the development of CC and assess the potential effects of modulating the VMB for the prevention and treatment of CC.

## Data Availability

The datasets used and/or analysed during the current study are available from the corresponding author on reasonable request.

## Abbreviations

CC: Cervical cancer
HPV: Human papillomavirus
HBV: Hepatitis B virus
HCV: Hepatitis C virus
EBV: Epstein-Barr virus
BV: Bacterial vaginosis
VMB: Vaginal microbiota
CIN: Cervical intraepithelial neoplasia
